# OTUD6A Is an Aurora Kinase A-Specific Deubiquitinase

**DOI:** 10.3390/ijms22041936

**Published:** 2021-02-16

**Authors:** Hyo Jin Kim, Jongchan Kim

**Affiliations:** Department of Life Sciences, Sogang University, Seoul 04107, Korea; khj19107@sogang.ac.kr

**Keywords:** Aurora kinase A, cancer, deubiquitinase, OTUD6A

## Abstract

Aurora kinases are serine/threonine kinases required for cell proliferation and are overexpressed in many human cancers. Targeting Aurora kinases has been a therapeutic strategy in cancer treatment. Here, we attempted to identify a deubiquitinase (DUB) that regulates Aurora kinase A (Aurora-A) protein stability and/or kinase activity as a potential cancer therapeutic target. Through pull-down assays with the human DUB library, we identified OTUD6A as an Aurora-A-specific DUB. OTUD6A interacts with Aurora-A through OTU and kinase domains, respectively, and deubiquitinates Aurora-A. Notably, OTUD6A promotes the protein half-life of Aurora-A and activates Aurora-A by increasing phosphorylation at threonine 288 of Aurora-A. From qPCR screening, we identified and validated that the cancer gene *CKS2* encoding Cyclin-dependent kinases regulatory subunit 2 is the most upregulated cell cycle regulator when OTUD6A is overexpressed. The results suggest that OTUD6A may serve as a therapeutic target in human cancers.

## 1. Introduction

Aurora kinases are serine/threonine kinases required for cell proliferation. There are three human paralogues; Aurora kinase A (Aurora-A), Aurora kinase B (Aurora-B), and Aurora kinase C (Aurora-C). Chromosomal missegregation during cell division causes genomic instability, contributing to carcinogenesis [1], and Aurora kinases play a role in proper chromosomal segregation. Aurora kinases are overexpressed in many human cancers including colorectal cancer, glioma, breast cancer, ovarian cancer, and pancreatic cancer [2,3,4,5,6,7], which has been driving an extensive investigation on their molecular functions in the cancer field and their roles as cancer targets in various human cancers. Among the three Aurora kinases, Aurora kinase A encoded by *AURKA* is a critical regulator in mitosis and meiosis and it is the most activated during the G2-M phase [8]. Aurora-A helps spindle body development [9], completes centrosome formation and segregation [8], and organizes and aligns chromosomes in prometaphase [10]. Aurora-A is often dysregulated in many cancer types. In breast cancer, in particular, it is overexpressed in 94% of cancer tissues [9]. Aurora-A has been shown to function as an oncogene, as evidenced by that it causes genomic instability in multiple cancers [11,12,13], inhibits apoptosis [14,15], promotes migration, invasion, and metastasis of cancer cells by controlling epithelial-mesenchymal transition (EMT), and maintains cancer stem cell properties [16,17]. Aurora kinases are activated in the actively dividing cells, which enables the distinction between cancer cells and rarely dividing normal cells. Therefore, targeting Aurora kinases is potentially an effective therapeutic strategy by specifically eradicating cancer cells. Therefore, small-molecule inhibitors targeting Aurora kinases including Aurora-A have been developed and tested in pre-clinical or clinical trials [18,19].

Ubiquitin is a small 76-amino acid protein and modifies proteins post-translationally through ubiquitination, a process of covalent attachment of ubiquitin to a substrate protein via lysine residues. Deubiquitination, on the other hand, reverses the reaction by removing the ubiquitin or ubiquitin chain from the substrate proteins. Aurora-A is well-known to be ubiquitinated by the APC/C E3 ligase at the end of mitosis, which facilitates its protein degradation through proteasome [20]. However, the deubiquitinase (DUB) that specifically deubiquitinates Aurora-A is not clearly identified yet. Therefore, if Aurora-A-specific DUB is identified, we can delineate the ubiquitination-deubiquitination process of Aurora-A, which allows us to better understand the cell cycle machinery regulated by Aurora A, a pivotal cell cycle regulator during the mitosis. Moreover, in cancer therapy, we can possess an alternative cancer therapeutic strategy in addition to directly targeting Aurora-A.

In the present study, we aimed at finding a DUB specific to Aurora-A using the human DUB library and identified OTUD6A. OTUD6A (OTU domain-containing protein 6A) is a member of Ovarian tumor-associated proteases (OTUs) comprising sixteen DUB members [21]. The tumorigenic roles of OTUD6A have been demonstrated previously. OTUD6A enhances the protein levels of Snail, an EMT (epithelial-mesenchymal transition) inducer [22], and promotes tumorigenecity by deubiquitinating and stabilizing Drp1 [23]. We found that OTUD6A deubiquitinates Aurora-A to stabilize and activate by interacting through the kinase domain of Aurora-A. Moreover, OTUD6A induces the transcription of an oncogenic cell cycle-regulating gene, *CKS2* (Cyclin-dependent kinases regulatory subunit 2) [24]. Thus, our study implicates the possibility of OTUD6A as a potential cancer therapeutic target.

## 2. Results

### 2.1. Identification of Aurora-A-Interacting Deubiquitinases (DUBs)

**Figure 1:**
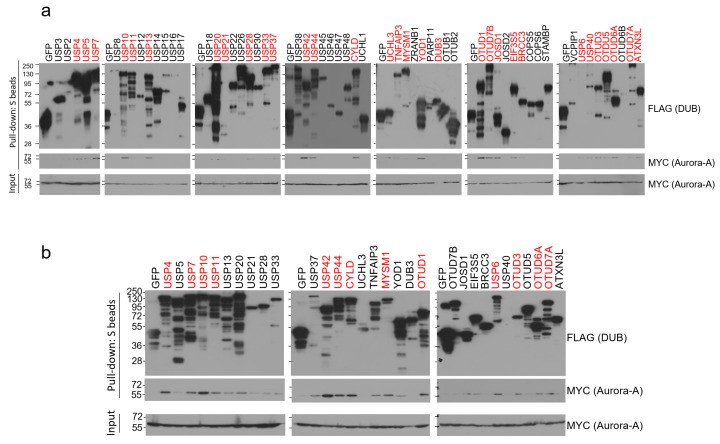
Pull-down assays to identify Aurora-A-binding DUBs. (**a**) Each SFB-tagged DUB was co-transfected with MYC-tagged Aurora-A into HEK293T cells, followed by pulling down DUBs with S-protein beads and immunoblotting with antibodies against FLAG (to detect DUBs) and MYC (to detect Aurora-A). The first screening identified 31 binding DUBs (highlighted in red). (**b**) Pull-down assay was repeated with 31 binding DUBs identified from the first screening. 13 DUBs (highlighted in red) which display a relatively strong binding affinity to Aurora-A were selected in this second screening.

To identify Aurora-A-specific deubiquitinase(s), we conducted binding assays using the human DUB library. We cloned a total of 85 human DUB open reading frames (ORFs) to the expression vector with SFB tag (a triple-epitope tag containing S-protein, FLAG tag, and streptavidin-binding peptide) and checked their expression by western blotting. Sub-library including 59 DUBs with decent expression in HEK293T cells was chosen and implemented for the pull-down assays. We transiently co-transfected each SFB-tagged DUB and MYC-tagged Aurora-A plasmids into HEK293T cells and pulled down DUBs with S-protein beads. As shown in Figure 1a, about half of the DUBs (31 DUBs) interacted with Aurora-A when immuno-blotted with MYC antibody. Their interactions were compared again in the second binding assays and 13 DUBs (USP4, USP7, USP10, USP11, USP42, USP44, CYLD, MYSM1, OTUD1, USP6, OTUD3, OTUD6A, and OTUD7A) showed relatively strong interaction with Aurora-A (Figure 1b). Previously, USP2 was shown to bind to and deubiquitinate Aurora-A [25], but we could not detect their interaction in our initial binding assays (Figure 1a). Therefore, USP2 was excluded from our candidates.

### 2.2. OTUD6A Deubiquitinates Aurora-A

**Figure 2:**
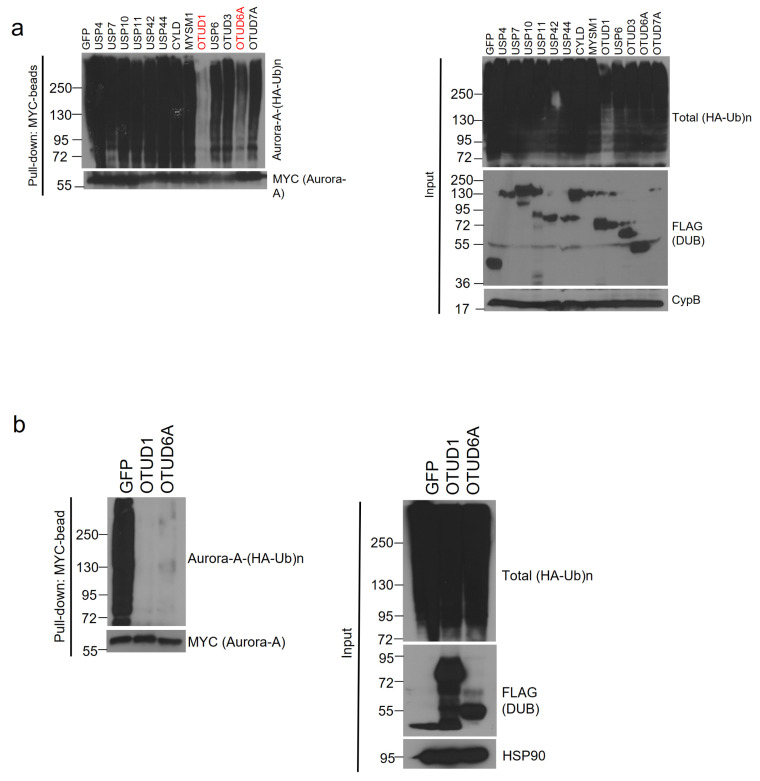
OTUD1 and OTUD6A deubiquitinate Aurora-A. (**a**) SFB-tagged 13 candidate DUBs were co-transfected with MYC-tagged Aurora-A and HA-tagged ubiquitin into HEK293T cells. After treating MG132 for 6 h, cells were lysed, followed by pulling down Aurora-A with MYC-beads and immunoblotting with antibodies against HA (to detect polyubiquitinated Aurora-A) and MYC (to detect Aurora-A). DUB assay identified OTUD1 and OTUD6A as Aurora-A-deubiquitinating DUBs. (**b**) DUB assay was repeated with OTUD1 and OTUD6A and a similar degree of deubiquitination of Aurora-A was observed.

Using the 13 Aurora-A-interacting DUBs, we conducted a deubiquitination assay. Each SFB-tagged DUB, MYC-tagged Aurora-A, and HA (hemagglutinin)-tagged ubiquitin plasmids were transiently co-transfected into HEK293T cells and the cells were treated with proteasome inhibitor, MG132. Aurora-A was immunoprecipitated with MYC antibody-conjugated agarose beads (MYC-beads). Only OTUD1 and OTUD6A induced the deubiquitination of Aurora-A (Figure 2a). Then, we conducted a deubiquitination assay again and found that both OTUD1 and OTUD6A similarly deubiquitinated Aurora-A (Figure 2b).

### 2.3. Aurora-A and OTUD6A Interact with Each Other through the Kinase and OTU Domains, Respectively

**Figure 3:**
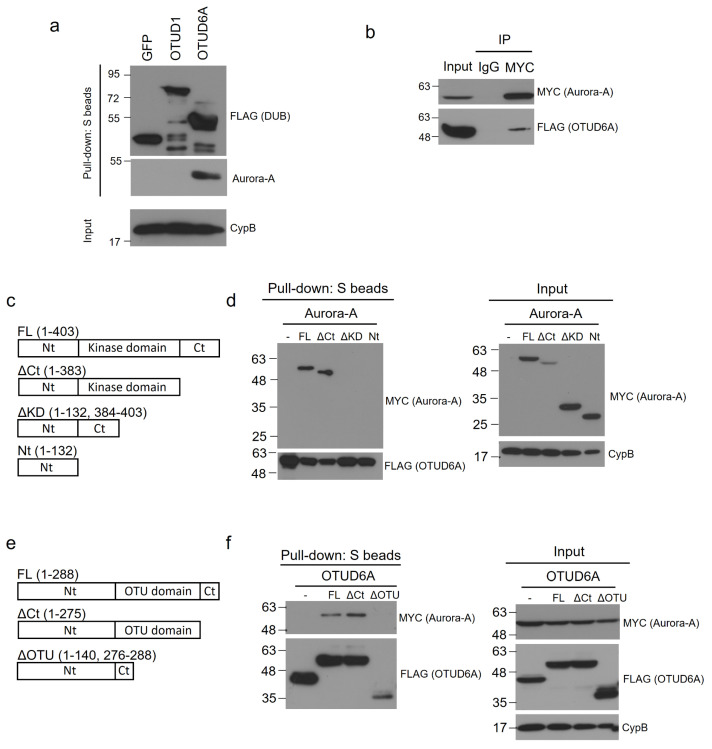
OTUD6A interacts with Aurora-A through the OTU and kinase domains, respectively. (**a**) SFB-tagged OTUD1 and OTUD6A were transfected into HEK293T cells, followed by pulling down DUBs with S-protein beads and immunoblotting with antibodies against FLAG (to detect DUBs) and Aurora-A. (**b**) SFB-tagged OTUD6A was co-transfected with MYC-tagged Aurora-A into HEK293T cells, followed by pulling down Aurora-A with MYC-beads and immunoblotting with antibodies against FLAG (to detect OTUD6A) and MYC (to detect Aurora-A). (**c**) Schematic representation of full-length (FL) and truncated mutants of Aurora-A. (**d**) SFB-tagged OTUD6A was co-transfected with MYC-tagged full length or each mutant Aurora-A into HEK293T cells, followed by pulling down OTUD6A with S-protein beads and immunoblotting with antibodies against MYC (to detect Aurora-A (FL and mutants)) and FLAG (to detect OTUD6A). (**e**) Schematic representation of full-length (FL) and truncated mutants of OTUD6A. (**f**) MYC-tagged Aurora-A was co-transfected with SFB-tagged full-length or each mutant OTUD6A into HEK293T cells, followed by pulling down OTUD6A (FL and mutants) with S-protein beads and immunoblotting with antibodies against MYC (to detect Aurora-A) and FLAG (to detect OTUD6A (FL and mutants)).

Next, we examined the interaction between endogenous Aurora-A and SFB-tagged OTUD6A. We transiently transfected SFB-tagged OTUD6A plasmid into HEK293T cells and pulled down OTUD6A with S-protein beads. Then, associated endogenous Aurora-A was blotted with Aurora-A-specific antibody. In this binding assay, Aurora-A exhibited a strong association with OTUD6A but weak or no interaction with GFP control protein and OTUD1 (Figure 3a). Based on this result, OTUD6A was chosen as a final candidate DUB and subjected to the subsequent experiments.

To confirm the interaction between Aurora-A and OTUD6A, we conducted reverse co-immunoprecipitation by pulling down MYC-tagged Aurora-A from the transfected HEK293T cell lysate and subsequently immunoblotted SFB-tagged OTUD6A with FLAG antibody. As shown in Figure 3b, OTUD6A indeed interacted with Aurora-A.

To determine the Aurora-A domain(s) that OTUD6A interacts with, we generated three truncated mutants of Aurora-A; Aurora-A^ΔCt^ (C-terminal-deleted, aa 1–383), Aurora-A^ΔKD^ (kinase-domain-deleted, aa 1–132 & 384–403), and Aurora-A^Nt^ (N-terminal only, aa 1–132) (Figure 3c). With the Aurora-A mutants, we conducted a binding assay with OTUD6A. We transiently co-transfected SFB-tagged OTUD6A and each MYC-tagged truncated Aurora-A mutant plasmid along with full-length wild-type Aurora-A^FL^ (aa 1–403) into HEK293T cells and pulled down OTUD6A with S-protein beads. As shown in Figure 3d, Aurora-A^FL^ and Aurora-A^ΔCt^ interacted but Aurora-A^ΔKD^ and Aurora-A^Nt^ did not interact with OTUD6A, which implies that OTUD6A binds to the kinase domain (KD) of Aurora-A.

Next, we examined which domain(s) on OTUD6A interact(s) with Aurora-A. We generated two truncated mutants of OTUD6A: OTUD6A^ΔCt^ (C-terminal-deleted, aa 1–275) and OTUD6A^ΔOTU^ (OTU-domain-deleted, aa 1–140 & 276–288) (Figure 3e). With the OTUD6A mutants, we conducted a binding assay with Aurora-A. We transiently co-transfected MYC-tagged Aurora-A plasmid and each SFB-tagged truncated OTUD6A mutant plasmid along with full-length wild-type OTUD6A^FL^ (aa 1–288) into HEK293T cells and pulled down each OTUD6A with S-protein beads. As shown in Figure 3f, OTUD6A^FL^ and OTUD6A^ΔCt^ interacted but OTUD6A^ΔOTU^ did not interact with Aurora-A, which implies that Aurora-A binds to the OTU domain of OTUD6A.

### 2.4. OTUD6A Promotes Protein Stability and Kinase Activity of Aurora-A

**Figure 4:**
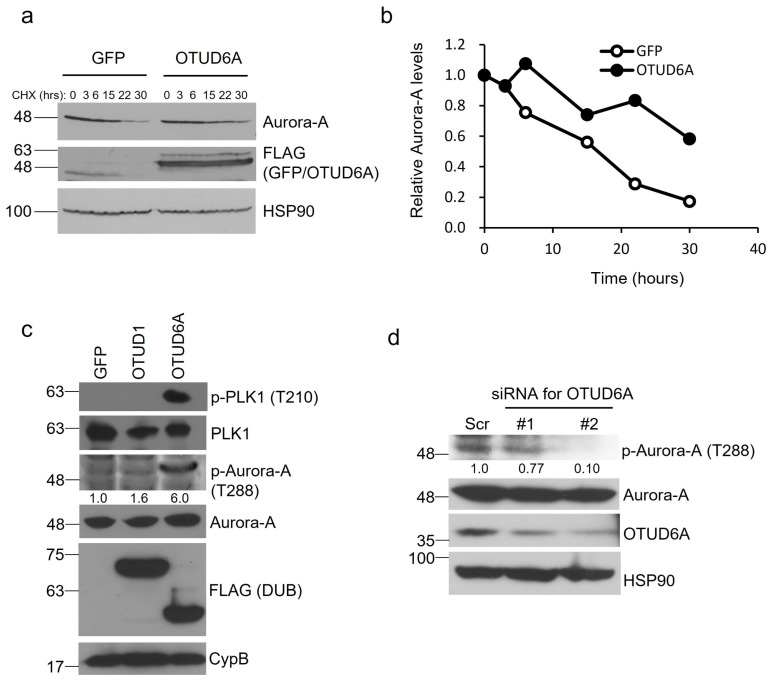
OTUD6A induces protein stability and enzymatic activity of Aurora-A. (**a**) SFB-tagged OTUD6A or GFP was transfected into HEK293T cells and translation inhibitor cycloheximide (50 μg/mL) was treated for the indicated period. The protein stability of endogenous Aurora-A was examined with a specific antibody. (**b**) Relative protein stability of Aurora-A tested in (**a**) was quantitated by normalizing with the expression levels of loading control HSP90. (**c**) The levels of phosphorylated Aurora-A at threonine 288 were induced by OTUD6A expression, which in turn increased phospho-PLK1 levels (at threonine 210). (**d**) Silencing OTUD6A with siRNAs suppressed Aurora-A phosphorylation at threonine 288.

To determine whether OTUD6A-mediated deubiquitination increases protein stability of Aurora-A, we transfected SFB-tagged OTUD6A into HEK293T cells and treated the cells with cycloheximide at 50 μg/mL for the indicated period. When normalized following quantitation, the protein half-life of Aurora-A was improved from ~17 to ~33 h by OTUD6A (Figure 4a,b).

Aurora-A is mainly activated after threonine 288 within the activation loop of the catalytic domain is autophosphorylated [26]. We examined whether OTUD6A promotes the kinase activity of Aurora-A by analyzing phosphorylation at threonine 288. When we overexpressed SFB-tagged OTUD6A in HEK293T cells, we could observe a lot more increase of phospho-Thr 288 by OTUD6A (+500%) than by OTUD1 (+60%) compared to the control. Moreover, activated Aurora-A by OTUD6A induced phosphorylation at threonine 210 of Polo-like kinase 1 (PLK1), a key substrate of Aurora-A, which is known to be a prerequisite for PLK1 activation in promoting mitotic entry [27,28] (Figure 4c). When we silenced OTUD6A with siRNAs, on the other hand, we could see 23–90% reduction in phospho-Thr 288 of Aurora-A (Figure 4d). Collectively, OTUD6A stabilizes Aurora-A and activates its enzymatic activity.

### 2.5. OTUD6A Promotes CKS2 Gene Expression

**Figure 5:**
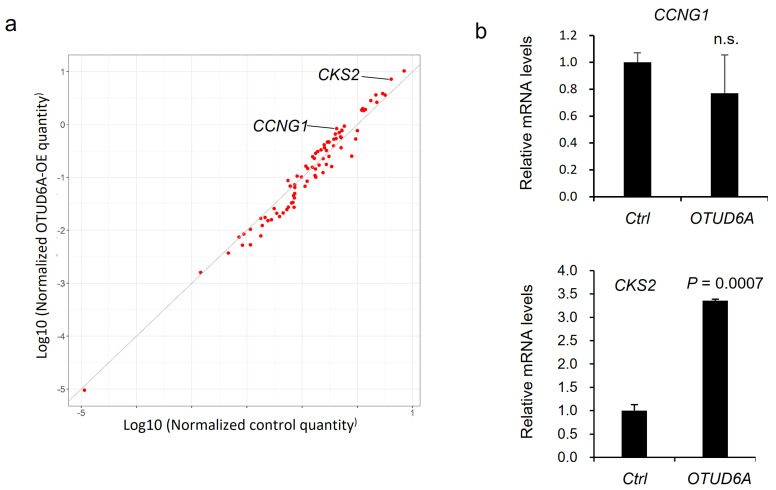
qPCR screening to identify cell cycle-regulating genes regulated by OTUD6A. (**a**) Scatterplot describes the relative expression of cell cycle-regulating genes in OTUD6A-overexpressing cells following qPCR screening. *CCNG1* and *CKS2* are indicated as two of the most upregulated genes. (**b**) qPCR validates *CKS2* expression is significantly upregulated by OTUD6A. Statistical significance in (**b**) was determined by an unpaired *t*-test. Error bars are s.e.m.

Aurora-A is an important cell cycle regulator during mitosis and is reported to regulate target gene transcription in a kinase activity-dependent manner. For example, Aurora-A affects target gene expression by stabilizing YAP transcription coactivator [29], and by phosphorylating at Serine 10 of histone H3 and the Runt-related transcription factors (RUNX) [30,31]. Therefore, we asked what cell cycle-regulating gene is the most upregulated by OTUD6A-mediated deubiquitination of Aurora-A. To address this question, we utilized qPCR screening kit which profiles cell cycle-regulating genes. Compared to the control, *CCNG1* (Cyclin G1) and *CKS2* (Cyclin-dependent kinases regulatory subunit 2) were two of the most upregulated genes in OTUD6A-transfected HEK293T cells in the screening (Figure 5a). We conducted an independent validation and found that *CKS2* was significantly upregulated by OTUD6A overexpression (Figure 5b).

## 3. Discussion

In the present study, we screened human DUBs that bind to and deubiquitinate Aurora-A in an unbiased manner. Previously, USP2 was identified as a DUB for Aurora-A but it did not show decent physical association with its substrate protein, Aurora-A in our screening, which prompted us to exclude it from the subsequent studies. OTUD1, one of the final candidate DUBs for Aurora-A, bound to and deubiquitinated Aurora-A but its interaction with endogenous Aurora-A was much weaker than OTUD6A. Moreover, OTUD1 was not able to increase the enzymatic activity of Aurora-A as much as OTUD6A. Therefore, OTUD6A was selected as a DUB that binds to, deubiquitinates, and activates Aurora-A. PLK1 is a key substrate of Aurora-A and phosphorylation at threonine 210 by Aurora-A occurs during G2 phase of the cell cycle, which is a prerequisite for PLK1 activation [27,28]. The activated PLK1 then phosphorylates substrates regulating mitotic entry such as CDC25C, promoting mitosis [32]. Consequently, our data suggest that Aurora-A, a critical mitotic regulator, is deubiquitinated, stabilized, and activated by OTUD6A, which in turn activates PLK1 to drive cells into mitosis.

In addition to PLK1, many Aurora-A downstream substrates have been identified and the functional roles of Aurora-A-mediated phosphorylation of the substrates have been extensively investigated in tumorigenesis [33]. For example, p53 is phosphorylated at multiple serine residues, which regulates its protein stability and transcriptional activity [34,35,36]. Phosphorylated Twist promotes epithelial-mesenchymal transition (EMT) and chemoresistance [37], and phosphorylated β-catenin becomes resistant to degradation and is promoted to localize into the nucleus, which results in increased transcriptional activity [38]. Phosphorylation of ERα (Estrogen receptor alpha) enhances its DNA-binding potential, which facilitates transcription of cyclin D1 [39]. Therefore, it is meaningful to examine whether such oncogenic pathways are also regulated by OTUD6A if OTUD6A is a bona fide DUB for Aurora-A.

We found that OTUD6A and Aurora-A interact with each other through their enzymatic domains, OTU and kinase domains, respectively. Moreover, Aurora-A is known to regulate target gene transcription by stabilizing YAP transcription coactivator [29], and by phosphorylating at Serine 10 of histone H3 and RUNX transcription factors [30,31], so we scrutinized how OTUD6A affects transcriptional programs by analyzing differentially expressed cell cycle-regulating genes. According to the qPCR screening, a cancer gene *CKS2* was validated as the most induced cell cycle regulator when OTUD6A was overexpressed. Cyclin-dependent kinases regulatory subunit 2 encoded by *CKS2* is known to regulate in vitro tumorigenecity of tongue squamous cell carcinoma, ovarian, cancer, esophageal cancer, breast cancer, and hepatocellular carcinoma [40,41,42,43,44]. One possible mechanism is that OTUD6A-mediated deubiquitination and stabilization of Aurora-A may increase YAP stability, which in turn activates *CKS2* transcription as a TEAD/YAP target gene. This idea is supported by the fact that the promoter region of *CKS2* genomic locus is bound by YAP according to the ChIP-seq analysis [45]. On the other hand, the expression of Aurora-A itself is directly regulated by transcription factors including FOXM1, ARID3A, E4TF1, and SIX3 [46,47,48,49] and non-DUB molecules such as Twist, YBX1, and ALDH1A1 have been shown to inhibit proteolysis of Aurora-A through the positive feedback loop with Aurora-A. However, the molecular mechanism by which Aurora-A is stabilized is unclear [37,50,51].

There are only a limited number of studies on OTUD6A. However, it has been demonstrated that OTUD6A enhances the protein levels of one of the crucial EMT inducers, Snail [22], and promotes colorectal cancer by deubiquitinating and stabilizing DRP1 [23]. Therefore, the present study and the previous reports suggest that OTUD6A could be a feasible therapeutic target in human cancers.

## 4. Materials and Methods

### 4.1. Cell Culture

The HEK293T cell line was obtained from American Type Culture Collection (ATCC) (Manassas, VA, USA). The cell line was cultured in Dulbecco’s Modified Eagle Medium (DMEM) supplemented with 10% fetal bovine serum (FBS) (GenDepot, Houston, TX, USA) at the humidified incubator with 5% CO_2_ at 37 °C.

### 4.2. Plasmids and siRNAs

Fifty-nine human DUB ORFs in Gateway Entry vector were obtained from DNASU Plasmid Repository (Manassas, AZ, USA) and DF/HCC DNA Resource Core (Boston, MA, USA) and were individually subcloned into a pBabe-SFB expression vector using the Gateway system (Thermofisher, Waltham, MA, USA). Aurora-A ORF in the Entry vector was subcloned into a MYC-tagged expression vector using the Gateway system. HA-ubiquitin vector was obtained from Addgene (Addgene #17608) (Watertown, MA, USA). siRNAs targeting OTUD6A were purchased from Genepharma (Shanghai, China) and their sense sequences are as followed: siRNA #1, GCUGGAGAAGUUCCAAGACTT; siRNA #2, GCACUACAACUCCGUGACATT.

### 4.3. Site-Directed Mutagenesis

We conducted mutagenesis to generate three Aurora-A and two OTUD6A truncated mutants with EZchange site-direct mutagenesis kit (Enzynomics, Daejeon, Korea) according to the manufacturer’s protocol.

### 4.4. Western Blotting

Western blotting was performed as described [52]. The cells were lysed in RIPA buffer supplemented with protease and phosphatase inhibitors (GenDepot, Houston, TX, USA). The proteins were separated by SDS-PAGE and transferred onto a PVDF membrane. The membranes were blocked in 5% non-fat milk in Tris-buffered saline-Tween 20 (TBS-T) and then incubated with the specific primary antibodies. After washed in TBS-T, the membranes were incubated with an HRP-conjugated secondary antibody. The bands were visualized by chemiluminescence. The following antibodies were used: antibodies against OTUD6A (1:1000, Proteintech, Rosemont, IL, USA), MYC (1:10,000, Proteintech, Rosemont, IL, USA), HSP90 (1:5000, Santa Cruz, Dallas, TX, USA), HA (1:1000, Santa Cruz, Dallas, TX, USA), FLAG (1:2000, Proteintech, Rosemont, IL, USA), Cyclophilin B (1:5000, Thermofisher, Waltham, MA, USA), Aurora-A (1:2000, Cell Signaling, Danvers, MA, USA), phospho-Aurora-A (Thr 288) (1:500, Abclonal, Woburn, MA, USA), PLK1 (1:1000, Abclonal, Woburn, MA, USA), and phospho-PLK1 (Thr 210) (1:1000, Cell Signaling, Danvers, MA, USA).

### 4.5. Immunoprecipitation and Pull-Down Assays

The cells were lysed in NETN buffer (200 mM Tris-HCl, pH 8.0, 100 mM NaCl, 0.05% Nonidet P-40, 1 mM EDTA) containing protease and phosphatase inhibitors (GenDepot, Houston, TX, USA). To pull down SFB-tagged proteins, cell lysates were incubated with S-protein beads (Merck, Darmstadt, Germany). To pull down MYC-tagged protein (Aurora-A), the cell lysates were incubated with MYC-beads (Thermofisher, Waltham, MA, USA) or normal mouse lgG-conjugated beads (Santa Cruz, Dallas, TX, USA). After inverting at 4 °C overnight, the precipitated protein complexes were washed three times with NETN buffer and the bound proteins were eluted by boiling in 2X Laemmli buffer and vortexing for 30 s and subjected to immunoblotting with the indicated antibodies.

### 4.6. Deubiquitination Assay

The HEK293T cells were co-transfected with MYC-tagged Aurora-A, SFB-tagged DUBs, and HA-tagged ubiquitin, and were treated with the proteasome inhibitor MG132 (10 μM) (Cayman, Ann Arbor, MI, USA) for 6 h. After cell lysis, MYC-tagged Aurora-A was immunoprecipitated with MYC-beads (Thermofisher, Waltham, MA, USA), and then polyubiquitinated Aurora-A was immunoblotted with HA antibody.

### 4.7. Total RNA Isolation, Reverse Transcription, and Quantitative PCR

Total RNA was extracted using MagListo RNA extraction kit (Bioneer, Daejeon, Korea) and RNase-free DNase I (Thermofisher, Waltham, MA, USA) and then reverse-transcribed with an iScript cDNA synthesis Kit (Bio-rad, Hercules, CA, USA). qPCR was performed using SYBR Green reagent (Enzynomics, Daejeon, Korea) and specific primer pairs. Real-time PCR and data collection were performed on a CFX Connect instrument (Bio-rad, Hercules, CA, USA). Gene expression was quantitated by the ΔΔCt method and was normalized to the expression levels of internal control (*β-Actin*). All qPCR reactions were performed in three replicates. Primer sequences used in the qPCR analysis are as followed; *CCNG1*-Forward, TCTTGCCTACGAGTCCCC; *CCNG1*-Reverse, GAGAGTCAGTTGTTGTCAGTACC; *CKS2*-Forward, CGCTCTCGTTTCATTTTCTGC; *CKS2*-Reverse, CTTGTTTGGAAAGTTCTCTGGG; *β-Actin*-Forward, AGGCACCAGGGCGTGAT; *β-Actin*-Reverse, GCCCACATAGGAATCCTTCTGAC.

### 4.8. qPCR Screening

qPCR screening kit for cell cycle-regulating genes was purchased from Bioneer (Daejeon, Korea). Gene list is available at https://www.bioneer.co.kr/20-sh-0001-10-cfg.html (accessed on 1 November 2020). Control or OTUD6A expression vector was transfected into HEK293T cells, and total RNA extraction and cDNA synthesis were performed as described above. 20 ng cDNA mixed with SYBR Green was distributed to each well of the 96-well format assay plate containing gene-specific primer sets. Real-time PCR and data collection were performed as described above.

### 4.9. Statistical Analysis

The data are presented as mean ± s.e.m., and a two-tailed unpaired *t*-test was used to compare two groups of independent samples. *p* < 0.05 was considered statistically significant.

## Data Availability

The data presented in this study are available upon reasonable request from the corresponding author.

## Abbreviations

SFB	A triple-epitope tag containing S-protein, FLAG tag, and streptavidin-binding peptide
DUB	Deubiquitinase
HA	Hemagglutinin
CypB	Cyclophilin B
GFP	Green fluorescent protein
HSP90	Heat shock protein 90
PLK1	Polo-like kinase 1
IP	Immunoprecipitation
IgG	Immunoglobulin G
FL	Full-length
Nt	Amino terminal
Ct	Carboxyl terminal
KD	Kinase domain
CHX	Cycloheximide
siRNA	Small interfering RNA
